# BAL for pneumonia prevention in tracheostomy patients: A clinical trial study

**DOI:** 10.12669/pjms.291.1971

**Published:** 2013

**Authors:** Amir K Vejdan, Maliheh Khosravi

**Affiliations:** 1 Amir K Vejdan, Assistant Professor of General Surgery, University of Medicine, Master of Burn and General Surgery Wards, Imam Reza Hospital, Birjand, Iran.; 2 Maliheh Khosravi, Resident of Anesthesiology, Department of Anesthesiology, Shariati Hospital, Tehran University of Medical Sciences, Tehran, Iran.

**Keywords:** Flexible Bronchoscopy, Bronchoalveolar Lavage, Pneumonia, Tracheostomy

## Abstract

***Objective:*** To evaluate the role of flexible bronchoscopy (FB) and bronchoalveolar lavage (BAL) on pneumonia prevention of tracheostomy patients in intensive care unit.

***Methodology: ***This clinical trial was conducted on 67 head-injury patients who needed tracheostomy. The eligible patients were divided into two groups of different methods for removing the airway secretions. In intervention group, FB and BAL was added to routine conventional methods for airway clearance. Patients were followed for signs and symptoms of pneumonia.

***Results*** *:* The risk of nosocomial pneumonia decreased from 35% to 14% in intervention group. The days of hospital stay were significantly reduced with bronchoscopic method.

***Conclusions:*** Flexible Bronchoscopy is recommended to all ICU admitted patients that have tracheostomy tube and high volume of secretion in their airways. It can not only prevent the pneumonia formation decrease the morbidity and mortality rate but it can even shorten the ICU stay time and consequently reduce the costs of treatment.

## Introduction

 Nosocomial infection is one of the main causes of death in hospitals.^1^^,^^2^ At present, nosocomial pneumonia and ventilator-associated pneumonia are the second leading cause of hospital-acquired infections and they have reported for about 10-15% of all nosocomial infections.^3^

 For unconscious head injury patients which required a tracheal tube for more than two weeks, it should be replaced by a tracheostomy tube. In this case pneumonia is formed by accumulation of secretions secondary to elimination of the cough mechanism.^4^ It is shown that any reduction in air way’s secretion make a remarkable decrease in incidence of pneumonia.^5^

 Drainage of the airways secretions which is normally done by cough mechanism, movement is one of the main physiologic mechanisms for prevention of nosocomial pneumonia. In intubated patients, this protective mechanism should be eliminated and airway’s secretions should be removed by aspiration from tracheal tube. The other ways for extraction of the secretions consist of: 1) humidification of the inhaled air by ventilators; 2) mucolytic and expectorants; 3) regular changing of the patient’s position; 4) percussion and chest physiotherapy; 5) tube suctioning; 6) regular changing of the tube; and 7) prophylactic antibiotics.^6^ The main disadvantage of these modalities is that they cannot extract the secretions completely especially mucous plaques. Thus, accumulation of these secretions inside the air ways, will finally lead to nosocomial pneumonia. Therefore, clearance of secretions under direct vision by bronchoscopy can remove all the secretions and mucous plaques.^7^

 Flexible bronchoscopy (FB), is a safe and accurate technique for assessment of the airways based on a direct vision, is mainly used as diagnostic purposes.^8^ In recent years this method has been used for clearance of pulmonary secretions in chronic obstructive pulmonary patients.^9^

 This study evaluates the role of FB and Bronchoalveolar lavage for pneumonia prevention of tracheostomy patients admitted in ICU.

## Methodology

 This prospective clinical randomized trial was carried out between 2007 and 2010 in the ICU of Imam Reza Educational Hospital, Birjand University of Medical Sciences, Iran. Prior to any procedure, informed written consent from legal relative were obtained. This study was approved by the Institutional Review Board of the Birjand University of Medical Sciences. Sixty seven head-injury adults who had high volume of secretions in their airways and a tracheostomy tube, were enrolled in this study.

 All selected patients were randomly divided into two groups including an interventional group and a conventional group using a computer-generated list. In the conventional group, patient secretions were managed with usual modalities and in the intervention group bronchoscopic cleaning was added to the routine methods.

***Conventional group (control): ***In this group we managed the patients’ airways secretion by following routine procedures: 1) gravity-assisted positioning like right lateral, left lateral and prone position; 2) vibrating and percussion were performed with hand or electrical instruments 3) humidification of inhaled air; 4) mucolytics and expectorants; 5) suctioning through the lumen of the tracheostomy tube with a plastic suction catheter.^10^^,^^11^

***Intervention group (technique of bronchoscopy): ***In the intervention group, the FB was used for clearance of airways in addition to the usual procedures. Ten minutes before starting the FB procedure, 10 ml of 2% lidocaine was injected into trachea trough the tracheostomy tube. In all our patients, bronchoscopic wash outs was performed by one surgeon in ICU with the cooperation of an anesthetist and an operation room nurse. A multichannel FB was used for irrigation and suction. For this purpose patients were placed in a semi-recumbent position (30 degrees). After a light sedation plus local anesthesia of airway, tracheostomy tube was removed and an early suction performed. Afterwards, the tip of bronchoscope was inserted into the patient’s airway through the opening of the tracheostomy. The bronchoscope was inserted into the trachea and then a full inspection was performed under direct vision. Watery secretions were simply suctioned; thick and adhesive secretions were first diluted by normal saline and then were suctioned. Previous studies indicated the role of instillation of normal saline before tracheal suctioning for prevention of pneumonia in mechanically ventilated patients.^12^^,^^13^ In some conditions, irremovable dried mucous plaques were removed by the bronchoscopic grasper. During the procedure, continuous pulse oxymetry and cardiac telemetry was performed and any decline in vital measures, led to temporarily stop of the procedure. All the secretions specimens were sent to laboratory for bacteriological examination, culture and antibiogram.

***Statistical Analysis: ***Statistical analyses were performed using SPSS v18 (SPSS, Chicago, Illinois, USA) and a *p* value less than 0.05 was considered significant. Results are given as means for continuous variables and number (percent) for categorical variables. Demographic and physiologic characteristics of the two groups were compared with use of Student’s t-test for continuous data and with the Mantel–Haenszel extended chi-square test for categorical data. Fisher’s exact test (two-tailed) was used when the expected number of cases per cell was below five.

## Results

 Sixty seven patients were enrolled in our study between June 2007 and September 2010. All of them had head injuries and underwent tracheotomy.

 Thirty-two patients were assigned to control group and 35 patients were in intervention group. The base-line characteristics of the two groups were similar (Table-I). The mean level of applied positive end-expiratory pressure was similar in both groups (5±2 cm of water in the both groups). Initial median GCS was similar for two groups as well (ranged between 3 and 9).

**Table-I:** Base-line characteristics of the patients.

*Variable*	*Bronchoscopic method* *(n=35)*	*Conventional method* *(n=32)*
Age (year)	28	29
Sex		
Male Female	19 (54.3%) 16 (45.7%)	17 (53.1%) 15 (46.9%)
Heart rate (beat/min)	85	79
Respiratory rate (breath/min)	16	15
Body tempreture (C)	37	38
Systolic blood pressure (mmHg)	120	122
Arterial pH	7.33	7.31
PaCO_2_ (mmHg)	41	40

 The frequency of clinically suspected nosocomial pneumonia was lower in the bronchoscopy group than the conventional group (five out of 35 [14%] vs. 11 out of 32 [34%]). The mortality rate related to the respiratory problems was 8.6% in the intervention group and 25% in control group (p<0.05). In BAL group only one patient had an infection of tracheostomy site whereas there were 10 cases (31%) with this infection in conventional group (p<0.05). The average nursing time per patient-day decreased from 54 to 8 minutes in bronchoscopic patients. However, care givers spent 46 more minutes per patient per day who was assigned to the control group (p<0.0001). The average length of ICU stay days for interventional and control patients was 12 and 18 days, respectively (p<0.0001).The cost of admission was decreased by 34% in the intervention group.

 Laryngospasm and bronchospasm (n=3), hypoxemia (n=5), cardiac arrthymias (n=2) were among the bronchoscopic related complications. All these side effects were transient and were managed by briefly stopping the procedure which was then completed without any complications. Only one sever COPD case could not tolerate the interventional procedure and the FB was stopped. No complication was seen during follow up time.

## Discussion

 Over the past decade diagnostic and therapeutic application of the optical technologies in medical procedures has greatly increased. The flexible bronchoscopy, as a well-known diagnostic and therapeutic method for improving the pulmonary function, has been used in our study for airways clearance in addition to the conventional modalities in ICU Admitted patients.

**Table-II:** Comparison between bronchoscopic and conventional methods.

	*Bronchoscopic method* *(n=35)*	*Conventional method* *(n=32)*	*p value*
ICU stay (day)	122	182	<0.0001
Patient-day nursing time (min.)	82	545	<0.0001
Nosocomial pneumonia	5 (14%)	11 (34%)	0.03
Tracheostomy site infection	1 (3%)	10 (31%)	0.001
Mortality related to respiratory problems	3 (8.6%)	8 (25%)	0.03

 The results show a significant decrease in the nosocomial pneumonia, mortality related to respiratory dysfunction, infection of tracheostomy site, length of ICU stay and the average nursing time altogether. In this context, BAL has been used mainly for diagnosis of pneumonia although; Scala et al introduced recently the FB as a safe and effective way for clearance of pulmonary mucous in chronic obstructive pulmonary disease as well.^7^^,^^10^ This innovative strategy was not associated with relevant complications, such as emergent intubation, cardiovascular events and pneumothorax thus these made it as a feasible and save method.^15^^-^^17^

 In this study, the incidence of nosocomial infection decrease in the intervention group significantly. The incidence of pneumonia in bronchoscopic and conventional methods was 14% and 34% respectively (p<0.0001). Terragni et al reported the prevalence pneumonia in mechanically ventilated adult ICU patients about 14 % and 21% in early tracheotomy late and tracheotomy respectively.^18^ Yavagal et al found that some pharmacological interventions may delay the development of nosocomial pneumonia but they did not decrease its frequency rate and had no effect on the mortality rate. The prevalence of pneumonia was 25% and 21% in placebo and Metoclopramide respectively.^19^ In recent decades many studies have been conducted for preventing the nosocomial infections in ICU admitted patients. These studies suggest that specific interventions can be employed to prevent pneumonia. These interventions focused on the prevention of aerodigestive tract colonization and the prevention of aspiration of contaminated secretions, mainly.^20^ The method used in this study can be categorized in both groups. The application of FB resulted in a significant decrease in volume of air ways’ secretions which finally lead to a decrease in the incidence of pneumonia. The results of other studies have shown that strategies concerning with reduction of the volumes and pathogenicity of aspirated material can prevent the pneumonia.^21^^,^^22^ The reported incidence of pneumonia in the other methods for prevention of pneumonia (including early and late tracheotomy, pharmacological treatment and conventional methods) is between 14% and 25%. The incidence of pneumonia in this study was 14% and 25% in intervention and control groups, respectively.^18^^,^^19^^,^^22^

 The results of this study show a dramatic decrease in the respiratory related mortality. The others studies have shown that nosocomial pneumonia independently contributes to increase the ICU patient’s mortality. Thus; any decrease in this infection resulted in reduction of mortality in ICU admitted patients.^23^ Among various preventive methods, the result of this study has proved that FB is an effective method for this purpose.^19^^,^^23^

 ICU stay and nursing time decreased in intervention group compared with the control group. This difference was statistically significant. The previous studies have shown that presence of pneumonia increased length of ICU stay and cost, spontaneously.^24^ The ICU stay was 12 (SD=±2) in this study. This finding is consistent with other studies that showed the mean length for the ICU stay in admitted pneumonia patients is about 9-12 days.^24^^,^^26^ On the other hand the results of this study show that application of FB decreases the incidence of pneumonia. As proved in other studies, any decrease in the incidence of pneumonia is accompanied with decrease in total cost.^27^ It also suggested that the costs for medical treatment gradually increase over time.^27^

 The main superiority of FB is its magnified direct vision of airways which can reveal any abnormality and remove it with the minimal invasive procedure. FB can remove the obstructions and take biopsy from a suspicious lesion. Electro coagulation can be used in case of minor bleeding too. One of the best features of FB is that this method is not dependent on the patient’s position and can be performed in any position. The centrifuged BAL specimen fluid can be used for bacterial culture and lead to administration of specific antibiotics.

## Conclusions

 We recommend BAL for airways clearance for all of the ICU admitted patients with tracheostomy tube and high volume of secretion. BAL can prevent the pneumonia formation and decrease the morbidity and mortality rate of ICU patients. This method can reduce the length of ICU stay which leads to decrease the total cost of treatment as well.

## Notes

***Conflicts of interest Notification Page: ***All authors of this paper declare any conflict of interest
